# RAD50, an SMC family member with multiple roles in DNA break repair: how does ATP affect function?

**DOI:** 10.1007/s10577-008-9018-6

**Published:** 2009

**Authors:** Eri Kinoshita, Eddy van der Linden, Humberto Sanchez, Claire Wyman

**Affiliations:** Department of Cell Biology and Genetics, Erasmus University Medical Center, Box 2040, 3000 CA Rotterdam, The Netherlands; Department of Cell Biology and Genetics, Erasmus University Medical Center, Box 2040, 3000 CA Rotterdam, The Netherlands; Department of Cell Biology and Genetics, Erasmus University Medical Center, Box 2040, 3000 CA Rotterdam, The Netherlands; Department of Cell Biology and Genetics, Erasmus University Medical Center, Box 2040, 3000 CA Rotterdam, The Netherlands. Department of Radiation Oncology, Erasmus University Medical Center, Box 2040, 3000 CA Rotterdam, The Netherlands. Department of Cell Biology and Genetics, Erasmus MC, PO Box 2040, 3000 DR Rotterdam, The Netherlands

**Keywords:** SMC protein, MRN complex, cohesin, condensin, double-strand break repair

## Abstract

The protein complex including Mre11, Rad50, and Nbs1 (MRN) functions in DNA double-strand break repair to recognize and process DNA ends as well as signal for cell cycle arrest. Amino acid sequence similarity and overall architecture make Rad50 a member of the structural maintenance of chromosome (SMC) protein family. Like SMC proteins, Rad50 function depends on ATP binding and hydrolysis. All current evidence indicates that ATP binding and hydrolysis cause architectural rearrangements in SMC protein complexes that are important for their functions in organizing DNA. In the case of the MRN complex, the functional significance of ATP binding and hydrolysis are not yet defined. Here we review the data on the ATP-dependent activities of MRN and their possible mechanistic significance. We present some speculation on the role of ATP for function of the MRN complex based on the similarities and differences in the molecular architecture of the Rad50-containing complexes and the SMC complexes condensin and cohesin.

## Introduction

The structural maintenance of chromosome (SMC) proteins, as their name implies, are involved in organizing DNA to assure proper function of chromosomes. Stated in a simplified way, condensins arrange and organize DNA within chromosomes and cohesins organize and arrange DNA between different chromosomes. SMC family proteins also have important roles organizing DNA for repair. DNA damage, such as double-strand breaks (DSBs), can disrupt chromosome architecture. DNA damage repair, especially repair of DSBs, is an essential element of maintaining or re-establishing proper DNA arrangement in chromosomes. The SMC family member Rad50, in complex with Mre11 and Nbs1, is a required component of DSB repair. Here we introduce the DNA repair functions of Rad50-containing complexes and compare their architecture with the SMC complexes cohesin and condensin. ATP binding and hydrolysis are important for all of these protein complexes. We review what is known about the molecular architectural effects and functional significance of ATPase activity for these proteins and highlight unresolved issues. The accumulated data suggest testable ideas about the architectural changes in the Rad50 complex and how these may be linked to ATPase activity.

## Double-strand break repair

DNA double-strand breaks (DSBs) are one of the most damaging occurrences for an organism. All organisms, therefore, have evolved intricate pathways to efficiently and systematically repair these breaks. Unrepaired DSBs can cause cell-cycle checkpoint arrest, ultimately leading to cell death. Improper repair can cause genome rearrangements, which in multicellular organisms are a common precursor to cancer. Eukaryotes have two main DSB DNA repair mechanisms: nonhomologous end-joining and homologous recombination. Nonhomologous end-joining rejoins DNA breaks with little or no homology, often resulting in deletions and insertions in the genome. Some breaks are directly ligated or joined after minimal processing. These sequences are aligned and the remaining DNA is removed via a nuclease or filled in by a DNA polymerase and then re-ligated (Weterings and van Gent 2004). Homologous recombination, in contrast, is an error-free, ‘accurate’ genetic recombination pathway that predominates in the S and G_2_ phases as it uses the undamaged homologous duplex as a template for repair synthesis (Wyman et al. 2004).

Homologous recombination requires DNA processing by architectural, structural and enzymatic factors. Homologous recombination begins with the recognition of the DSB followed by 5′ to 3′ nuclease processing which yields 3′ single-stranded DNA (ssDNA). This 3′-ssDNA is covered by RPA (replication protein A), which is subsequently displaced by the DNA strand exchange protein Rad51. Assembly of Rad51 onto properly processed ssDNA is aided by recombination mediators such as Rad54, Rad54B, Rad50/Mre11, the Rad51 paralogues (XRCC2, XRCC3, Rad51B, Rad51C, and Rad51D), and BRCA2 (Symington 2002; Wyman et al. 2004; Wyman and Kanaar 2006). Rad51 forms a nucleoprotein filament on ssDNA that invades homologous DNA segments for eventual polymerase-mediated extension (West 2003). The process is then completed with strand resolution and ligation. DSB repair pathways are closely linked to cell-cycle checkpoint signaling via the ATM checkpoint kinase (D’Amours and Jackson 2002; Assenmacher and Hopfner 2004). ATM activation causes cell-cycle arrest until the DNA breaks are repaired or cells undergo apoptosis (Khanna and Jackson 2001).

## Multiple roles of the MRN complex in DSB repair

**Fig. 1:**
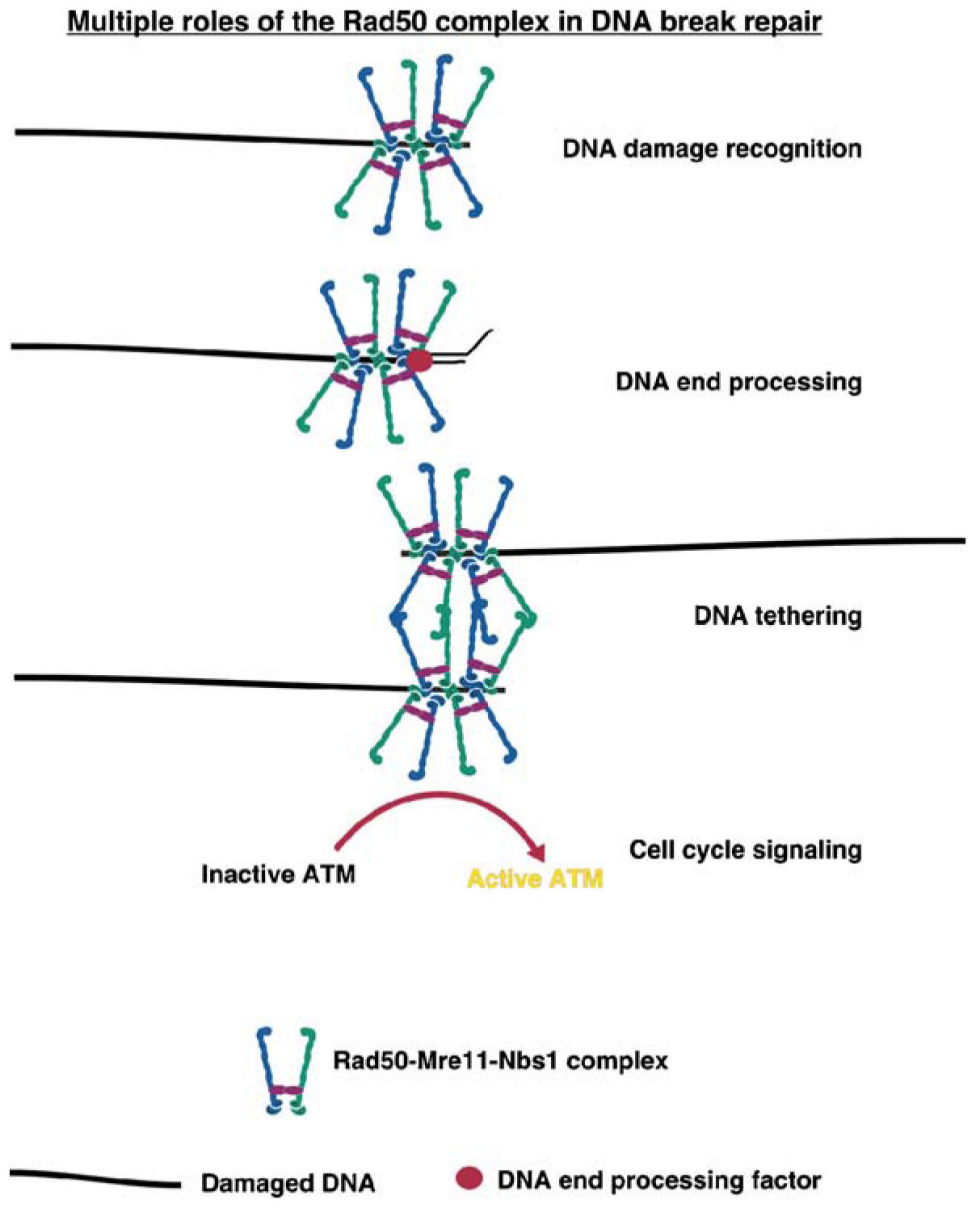
The multiple roles of Rad50 complexes in DNA break repair are illustrated. From top to bottom: Rad50 complexes bind to DNA early in the repair process to recognize double-strand breaks. Multiple Rad50 complexes bind to DNA. Rad50 complexes are involved in DNA processed including strand unwinding and nuclease digestion. This involves additional components that have not yet been clearly defined in all systems. DNA ends bound by Rad50 complex multimers are tethered by interaction among multiple coiled-coil apex hook domains. ATM is activated for cell-cycle signaling by interaction with DNA-bound Rad50 complexes; this step requires the Nbs1 component

The MRN complex is involved at several distinct steps in DSB repair including break recognition, DNA end processing, and signaling for cell cycle arrest (Fig. 1). The MRN complex is a primary damage sensor involved in the early steps of DSB repair in both human and yeast cells (D’Amours and Jackson 2002). The importance of Rad50, Mre11, and Nbs1 genes in mammals is illustrated by the cell nonviability or embryonic lethality resulting when any of these three genes is disrupted (Xiao and Weaver 1997; Luo et al. 1999; Zhu et al. 2001). In humans, mutations in Nbs1 cause Nijmegen breakage syndrome (NBS). NBS patients show radiation sensitivity, immune system deficiency, and a high rate of malignancy (Shiloh 1997). NBS patients show phenotypes similar to ataxia-telangiectasia (A-T), a related radiation sensitivity disorder. A-T is caused by mutations in the A-T mutated gene (ATM), which encodes a large protein kinase that initiates DNA damage signaling in response to DSBs. A connection between MRN and ATM arose with the identification of two families with A-T-like disorder (ATLD), clinically identical to A-T but caused by mutations in Mre11 (Stewart et al. 1999).

The link between MRN recognition of DNA damage and activation of ATM cell-cycle checkpoint signaling is also supported by biochemical data. ATM activation is suggested to involve two steps, first recruitment of ATM dimers to sites of DNA damage where they dissociate into monomers then activation of ATM monomers to become functional kinases (Dupre et al. 2006). ATM then phosphorylates many proteins important for the DNA damage response. Nbs1 is required to activate ATM and is also a substrate for ATM kinase activity (Lee and Paull 2004; Paull and Lee 2005). The MRN complex has additional functional and physical interactions with other DNA repair and cell-cycle checkpoint proteins (reviewed in Assenmacher and Hopfner 2004).

## Structure and function of MRN components

All DSB repair functions of MRN involve interactions with DNA that require at least Rad50 and Mre11. Homologues of Rad50 and Mre11 exist in archaea, fission and budding yeasts, as well as higher metazoans (Hopfner et al. 2000a; D’Amours and Jackson 2002). The Mre11-Rad50 (MR) complex has essential functions early in DSB repair, based both on time of accumulation at breaks in cells and its biochemical activities (Assenmacher and Hopfner 2004; Lisby et al. 2004). The associated Nbs1 (also known as nibrin) or Xrs2 proteins (MRN or MRX complexes), in mammalian and yeast cells, respectively, link the Mre11-Rad50 complex to cell-cycle checkpoint activation (Assenmacher and Hopfner 2004).

**Fig. 2:**
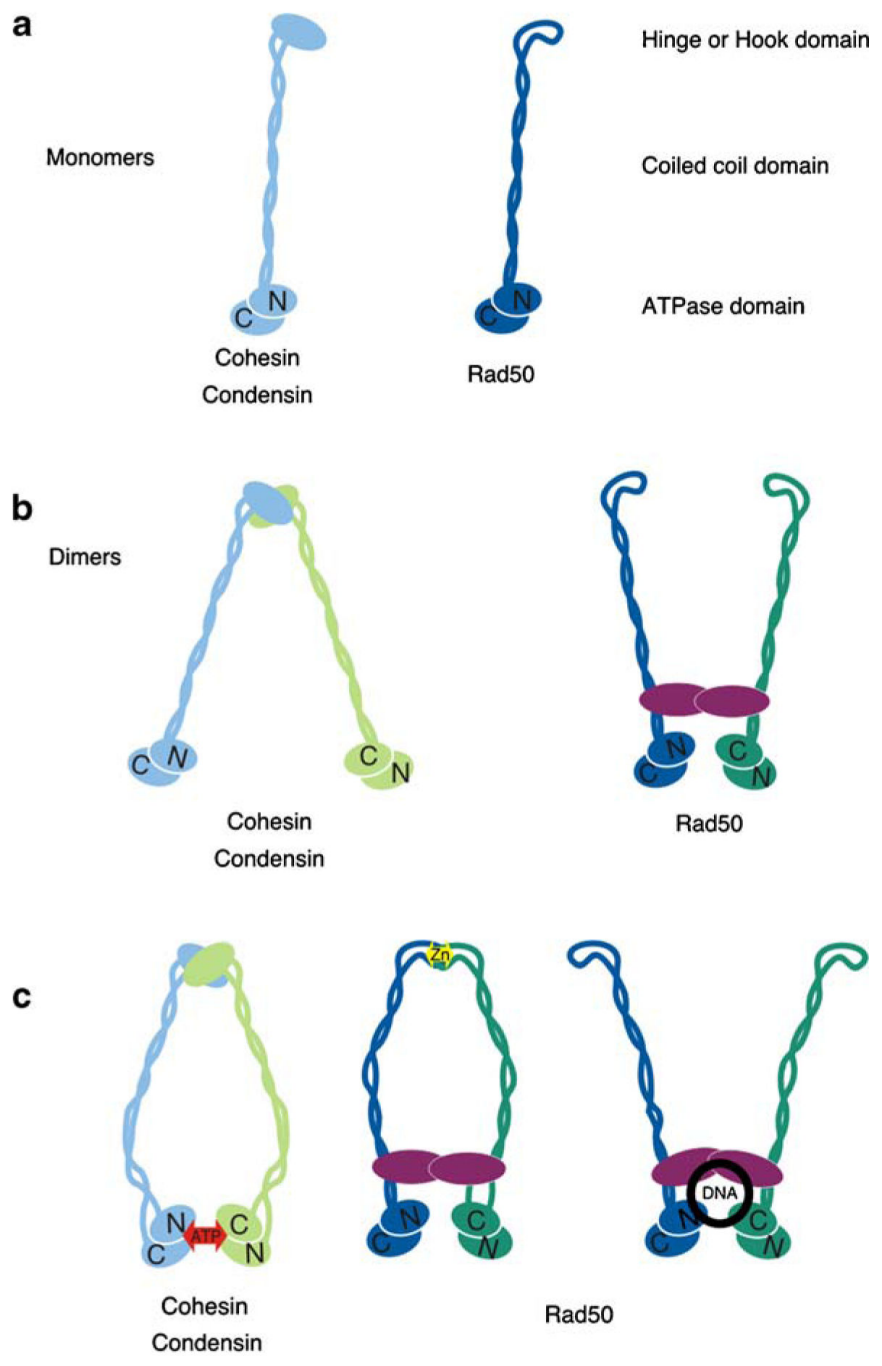
Comparison of the molecular architecture of SMC proteins (generalized for condensin and cohesin) and Rad50. **a** The arrangement of protein domains in cohesin/condensin and Rad50 monomers. The N- and C-terminal amino acid domains are juxtaposed at one end of an intramolecular coiled coil. This constitutes an ATPase head domain. The apex of the coiled coil where it folds back on itself is a globular dimerization domain for condensin and cohesin called the hinge. For Rad50 the coiled-coil apex is a smaller CxxC amino acid motif, called the hook; **b** The arrangement of SMC and Rad50 proteins in dimers. For condensin and cohesin, two elongated monomers are held together by a stable dimer interface between hinge domains. For Rad50 complexes, an Mre11 dimer binds two elongated Rad50 monomers, holding them together by interaction along the coiled coils near the ATPase heads; **c** Additional interactions among complex components. ATP binding occurs at the interface of two ATPase monomers. In this simplified cartoon for condensin and cohesin, this results in joining of the coiled coils at both ends, forming a large protein ring. Rad50 complexes are held together by Mre11 but can additionally interact at the coiled-coil apexes. Two CxxC hook domains can coordinate a zinc ion and cause a similar large protein ring to form. However, if DNA is bound at the globular ATPase/Mre11 end of the complex, the arrangement of the coiled coils changes so that they no longer interact with each other within the same complex

Rad50 resembles the SMC proteins involved in chromosome cohesion and chromatin condensation (Aravind et al. 1999; Strunnikov and Jessberger 1999). SMC proteins all contain Walker A and B nucleotide (NTP)-binding motifs at their amino- and carboxy-terminal ends, respectively. These motifs are separated by long stretches of amino acids that form an extended coiled-coil structure. The coiled coils fold back on themselves to form intramolecular association of the ATPase domains at one end and a so-called hook or hinge domain at the other end of an elongated structure (Fig. 2a). These structural elements and their architectural arrangement are exploited for various functions of Rad50 and related proteins. The core Rad50 complex is a heterotetramer of Mre11 and Rad50 (M_2_R_2_) arranged such that the DNA-binding sites on the Mre11 dimer are close to the two Rad50 ATPase domains (Fig. 2b) (de Jager et al. 2001b; Hopfner et al. 2001). The Mre11/Rad50 (MR) coiled coils are notably flexible (de Jager et al. 2001b; van Noort et al. 2003; Moreno-Herrero et al. 2005). RM binds to DNA via the globular domain with the coiled coils protruding (de Jager et al. 2001b). DNA is an allosteric effector of the RMN complex as binding DNA induces reorientation of the RAD50 coiled coils to become parallel to one another favoring the inter-complex interactions needed for DNA tethering and organizing DNA for eventual repair (Fig. 2c) (Moreno-Herrero et al. 2005).

Mre11 orthologues have a number of enzymatic activities that may play important roles in DNA end processing for eventual repair. Activities identified *in vitro* include: Mn^2+^-dependent ssDNA endonuclease, double-strand DNA (dsDNA) 3′ to 5′ exonuclease, and DNA hairpin opening enzymatic activities (Paull and Gellert 1998). In this study, both exonuclease and hairpin opening activities are stimulated by Rad50 and ATP. X-ray structures of *P. furiosus* Mre11 showed that the catalytically active center of the protein has two domains. Domain 1 consists of the N-terminal calcineurin-like phosphoesterase with nuclease catalytic motifs and coordinates Mn^2+^. Domain 2 consists of C-terminal DNA-binding domains with potential Rad50-interacting domains (Hopfner et al. 2001). The structure of *P. furiosus* Mre11 bound to Mn^2+^ and a 5′-dAMP nucleotide hydrolysis product indicates that Mre11 has 3′ to 5′ directionality. This polarity is opposite that required to process double-stranded DNA ends into the 3′ single-stranded overhangs needed for the strand exchange step of homologous recombination.

*In vivo* a role for MRN in DNA end processing for homologous recombination would require reversing the directionality of the Mre11 nuclease or an additional 5′ to 3′ exonuclease. Mre11, Rad50, and Xrs2 in *S. cerevisiae* are involved in producing 3′-ssDNA from DSBs. This is based on the phenotype of Rad50 and Mre11 mutants, which stall during the initiation of meiosis because DSBs are formed but not resected (Alani et al. 1990). However, Mre11 is a 3′ to 5′ exonuclease with or without Rad50 (Paull and Gellert 1998), suggesting that other factors are involved in creating 3′ ends. Candidate factors have been identified as CtIP in mammals, Sae2 in *S. cerevisiae*, and Ctp1 in *S. pombe*, proteins that interact with Mre11 to promote DSB resection (Clerici et al. 2005; Lengsfeld et al. 2007; Limbo et al. 2007; Sartori et al. 2007).

Cell-cycle checkpoint signaling requires the third component in the MRN complex, Nbs1 (or its functional homologue Xrs2 in *Saccharomyces cerevisiae*) (D’Amours and Jackson 2002; Stracker et al. 2004). The Nbs1 protein family is less conserved and has so far been described only in mammals. On current evidence, including reduced Nbs1 association with MR complex including a mutant Mre11 and purification of a stable Mre11-Nbs1 complex, it is expected that Nbs1 associates with the complex via interaction with Mre11 (Stewart et al. 1999; Lee et al. 2003). However, the protein–protein interactions among the three components necessary for Nbs1 association and the architectural arrangement of Nbs1 with respect to the other components have not yet been determined. Because of its large overall mass and the very elongated form of Rad50, attempts to determine the stoichiometry of Nbs1 within the complex have so far not succeeded (Lee et al. 2003). Indeed, our current data (E. van der Linden. and C. Wyman, unpublished) is consistent with the remark of Lee et al. that ‘the triple complex may in fact be a collection of complexes with varying stoichiometry’.

## ATP modulates molecular architecture

**Fig. 3:**
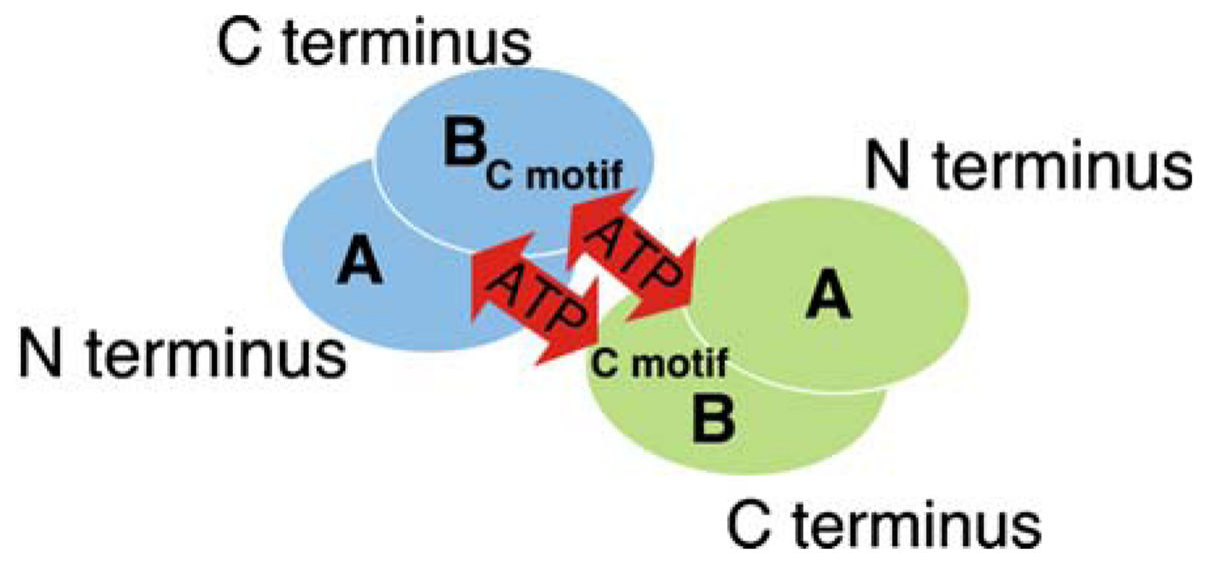
Illustration of the ATP binding sites at the interface of two ABC ATPase monomers. Only the globular ATPase domains of a generic ABC ATPase, here representing an SMC protein or Rad50, are shown. The Walker A and Walker B motifs are located at the N- and C-terminal ends of the protein, respectively. Domains of the same color are from the same protein or polypeptide chain. ATP binds at the dimer interface, whereby the Walker A and B nucleotide-binding domains contact a highly conserved signature motif (C motif) from a second protein. Two ATPs are shown as there are two possible binding sites formed in a dimer. However, it is not known whether two ATPs can or do bind simultaneously

ATP binding and hydrolysis cause architectural rearrangements in SMC proteins. A functional ATPase is formed in the characteristic SMC structure when intramolecular antiparallel coiled-coil interactions bring the N-terminal Walker A and C-terminal Walker B nucleotide-binding domains together (de Jager et al. 2001b; Haering et al. 2002; Hopfner et al. 2002). These nucleotide-binding domains place SMC proteins in the conserved family of ATP-binding cassette (ABC) ATPases (Hopfner and Tainer 2003; Ye et al. 2004). Although proteins in this family have diverse functions, their ATPase modules share structural and mechanistic properties. ATP binds at a dimer interface whereby the Walker A and B nucleotide-binding domains contact a highly conserved signature motif (C motif) from a second protein (Fig. 3) (Hopfner et al. 2000b). Current evidence supports a picture of functional SMC dimers whereby ATP binding to the two ATPase head domains triggers engagement of two subunits, and subsequent ATP hydrolysis leads to disengagement of this dimer interface (Figs. 2b and c) (Arumugam et al. 2003; Weitzer et al. 2003; Lu et al. 2005).

This ATPase cycle is well described for the *B. subtilis* SMC protein where specific mutations block the ATPase cycle at different stages (Hirano et al. 2001; Hirano and Hirano 2004, 2006). A mutation in the C motif that allows ATP binding but blocks ATP-driven dimer engagement also abolished ATP hydrolysis, supporting the idea that head–head engagement is essential for ATP hydrolysis. A so-called transition state mutant stabilizes the dimeric state by slowing down ATP hydrolysis. Similarly, the eukaryotic SMC1/3 complex of cohesin binds and hydrolyzes ATP at a dimer interface. There is added complexity here as the ATPase activity of cohesin is controlled by an additional subunit, Scc1, interacting with the ATPase domains of SMC1/3 (Arumugam et al. 2006). The ATPase cycle is common to SMC proteins and ABC transporters. How this ATP-dependent engagement–disengagement cycle facilitates the diverse functions of the different ABC ATPases has not yet been mechanistically defined. ATP hydrolysis is essential for the SMC complexes involved in chromosome segregation and condensation, but its specific role in enabling or modulating the several predicted DNA transactions is far from understood.

Bacteria have additional SMC family members involved in DNA repair whose function appears to be modulated by ATP. Sequence homology comparison shows that RecN and SbcC proteins have a similar organization to eukaryotic SMCs (SMC1/3, SMC2/4, SMC5/6, or Rad50) (Hopfner and Tainer 2003; Sanchez et al. 2008). Additionally, the crystal structure of a bacterial RecF protein, involved in homologous recombination DNA repair, shows a high degree of structural similarity with the ATPase head of RAD50 (Koroleva et al. 2007). The SbcC and SbcD together form a complex with an architectural arrangement similar to eukaryotic MR including a CXXC amino acid motif that defines the hook domain in the long coiled coil (Connelly et al. 1998; Connelly and Leach 2002). Reminiscent of the MR complex, purified SbcCD is an ATP-dependent double-stranded exonuclease and ATP independent single-stranded endonuclease, with nuclease activities that depend on Mn^2+^ (Connelly and Leach 1996; Connelly et al. 1997). Biochemical studies also point out several functional similarities between RecN and eukaryotic MRN. Although RecN is an ssDNA-dependent ATPase, it binds DNA independently of ATP. RecN forms large DNA networks with ssDNA or duplex molecules containing ssDNA regions in the presence of ATP or ADP (Sanchez and Alonso 2005; Sanchez et al. 2008). Similarly to Rad50, the primary sequence of RecN predicts that a functional ATP binding site can be formed by association of the N- and C-terminal Walker A and B motifs.

## The role of ATP in DNA binding by Rad50 complexes

The *B. subtilis* SMC protein provides some specific clues as to how ATP binding and hydrolysis may influence DNA binding in related proteins. *B. subtilis* SMC is a homodimer that can bind DNA via its hinge domain (Hirano and Hirano 2006). Two different forms of DNA binding modulated by the ATPase cycle have been described (Hirano and Hirano 2006). A less stable interaction with DNA, called the sitting mode, stimulates ATPase activity leading to head–head disengagement and opening of the proposed ring formed by the coiled coils, which are still held as a dimer via the hinge–hinge interface (Fig. 2b compared to Fig. 2c). This disengagement of the ATPase domain interface is a prerequisite for the formation of more stable DNA binding, called the hooking mode. In this more stable binding mode, ATP has a positive effect on DNA binding as head–head engagement can lead to the capture of a second DNA duplex within a ring formed when the coiled coils are connected at both hinge and ATPase ends. These ideas are conceptually similar to the ring model proposed for the function of eukaryotic cohesin complex (Haering et al. 2002), in which two DNA duplexes are held within a protein ring formed by association of the coiled coils at both hinge and ATPase head domains (Haering et al. 2008), although here specific DNA binding sites have not been defined. The *B. subtilis* SMC, like other SMCs, interacts via its ATPase domain with non-SMC proteins, in this case ScpA and ScpB (Hirano 2005). Binding of ScpA and ScpB suppresses the ATPase activity, thereby stabilizing engagement of the ATPase domains (Hirano and Hirano 2004).

Although the present models for the function of the several SMC members are still speculative, the above data show that the ATPase cycle is linked to changing interactions among the subunits and possibly regulates DNA binding in multiple ways. Various studies suggest that ATP binding or hydrolysis is important for MR(N) function. ATP likely acts as a structural switch that changes the conformation of MR(N). The addition of ATP, and more so AMP-PNP, increased the preference of purified human MR for forming large oligomers on DNA substrates with 3′-overhangs compared to blunt ends and 5′-overhangs (de Jager et al. 2002). Based on x-ray crystallography of isolated ATPase domains, ATP binding causes two major structural rearrangements to the ATPase domain of Rad50. Firstly, there is a 30° rotation of the C-terminal lobe relative to the N-terminal lobe; and secondly, the two ATPase motifs of Rad50 form into a compact homodimer (Hopfner et al. 2001). It was proposed that ATP-induced rotation repositions bound DNA with respect to Mre11 (Hopfner et al. 2001). The specific roles of the ATP-induced conformational changes within the context of the complete RMN complex are still to be elucidated.

A specific requirement for ATP in DNA binding by the MR(N) complex has been suggested in several studies, but not observed in others. Rad50 originally purified by itself from *S. cerevisiae* (Raymond and Kleckner 1993) bound DNA dependently on ATP in a filter-binding assay. Similarly, a protein construct including the N- and C-terminal ATPase domains of *P. furiosus* Rad50 (hereafter referred to as pfRad50cd for catalytic domain) dimerized in the presence of ATP, as determined by dynamic light scattering (Hopfner et al. 2000b). In this same study, an electrophoretic mobility shift assay (EMSA) showed that the same concentrations of pfRad50cd bound more DNA in the presence of ATP. However, purified complexes of human MR or MRN exhibited ATP-independent DNA binding in one study (Paull and Gellert 1999) but nucleotide and NBS1-dependent DNA binding in another (Lee et al. 2003). Amino acid substitutions in the conserved ATP binding signature motif of human RAD50 abolish DNA binding by the resulting MRN complex (Moncalian et al. 2004), also implying that ATP is required for DNA binding. However substantial DNA binding by human MR complex in the absence of added nucleotides was observed in EMSA assays as well by SFM imaging that demonstrated nucleotide-independent DNA binding, oligomerization, and tethering (de Jager et al. 2001b). A careful look at these different studies shows some important differences that may help clarify the apparently disparate results.

Binding of protein to a substrate, DNA in this case, is characterized by association and dissociation constants related to concentration. Various DNA binding studies, described above, report somewhat different behavior of Rad50-containing complexes. These reported differences may of course be due to varying conditions for the binding reactions and assays. However, any change in the implied affinity of protein for DNA, due to presence or absence of cofactors, would be due to differences in binding constants. The association constants for Rad50, or complexes including Rad50, binding to DNA have not been rigorously determined, and may be difficult to sort out for reasons described below. Nevertheless, apparent DNA-binding activity depends on the concentration of protein and DNA, and these factors differ among the published reports. Nucleotide-independent DNA binding is observed by EMSA and SFM imaging at MR protein concentrations in the range of 10–100 nM (de Jager et al. 2001b, 2002). Varying nucleotide-dependent binding is observed in studies where DNA substrates and protein complex are present in low nanomolar concentrations (Paull and Gellert 1999; Lee et al. 2003). Comparing these studies indicates that large differences in DNA binding behavior are observed with 2–3-fold changes in protein concentration. This may indicate that the *K*_d_ for MR or MRN binding to DNA is in the range of 10 nM. It is also possible that the purified proteins used in these studies may differ slightly in composition, quality, and specific activity.

In addition to considerations of the amount and quality of protein used, the type of DNA in the different assays may influence apparent binding affinity. EMSA assays typically use relatively short DNA, in the range of 50–160 nt or bp in the studies cited above, which would accommodate binding of one or a few MR(N) complex(es). The SFM imaging experiments use longer DNA in the range of 1–5 kbp. Binding of MR(N) to longer DNA substrates, on the order of 1 kbp, may involve protein–protein interaction in addition to protein–DNA interaction. SFM imaging shows that the longer DNA is bound by oligomeric assemblies in which protein–DNA complex formation and stability could be influenced by favoring interactions among proteins brought near each other by binding to DNA. The multiple DNA–protein and protein–protein interactions taking place on DNA substrates capable of binding multiple MR (N) complexes make it difficult to determine simple protein–DNA affinities and binding constants.

One way to interpret these reports of ATP-dependent and ATP-independent DNA binding by MR and MRN is that there are different DNA binding sites and that ATP effects access to or assembly of DNA-binding sites. This idea reflects the proposed different DNA binding modes and their control by ATP binding described for *B. subtilis* SMC (Hirano and Hirano 2006). For the MR complex, different DNA-binding sites are expected as separately both Mre11 and Rad50 bind DNA (Paull and Gellert 1998; Raymond and Kleckner 1993; de Jager et al. 2001a; Hopfner et al. 2000a). Whereas DNA binding by Mre11 is ATP independent, ATP is required for Rad50 alone to bind DNA. Thus the different effects of ATP on DNA binding by MR or MRN complexes may reflect binding to these two different sites. In addition, the access to DNA-binding sites in the protein complex may depend on ATP binding-induced changes in molecular architecture.

By analogy with well-described SMC proteins, it is expected that ATP binding to Rad50 will cause ATPase domains to engage as a dimers and ATP hydrolysis will cause disengagement. X-ray crystallographic studies of pfRad50 catalytic domain reveal a 30° rotation of domains relative to each other induced by ATP binding (Hopfner et al. 2000b). Interestingly, biochemical analysis of eukaryotic MR shows enhanced protein–DNA interaction with AMP-PNP (a nonhydrolyzable analogue of ATP) (Lee et al. 2003). These authors reasoned that AMP-PNP binding might block DNA release that is otherwise triggered by ATP hydrolysis. They suggest that the requirement for a nonhydrolyzable ATP analogue implies rapid ATP hydrolysis by MRN. However ATP turnover rates for MR are rather slow, ranging from 0.026 to 0.08 per min per MR complex (de Jager et al. 2002; Bhaskara et al. 2007). A clearer picture of the importance of ATP in Rad50 complex function will require further consolidation of the wealth of available data, as well as consideration of more subtle roles of nucleotide cofactors. For instance, possible effects of DNA binding on MR affinity for and exchange of bound nucleotides has not been determined, nor are the kinetics of ADP release well defined. These mechanistically interesting events are possibly linked to changes in protein complex architecture during the ATPase cycle. In addition, the discussion we present here predicts new aspects of MR(N) function in organizing DNA that could be controlled in the ATPase cycle. These structure–function connections include: (1) the existence of different binding sites or modes in the MR(N) complexes, (2) the importance of protein–protein interactions for controlling DNA binding, and (3) architectural changes in MR(N) that could influence inter- or intra-complex contacts and subsequently control formation of or access to DNA-binding sites.

## Rad50 and SMC complexes; similarities and differences

**Fig. 4:**
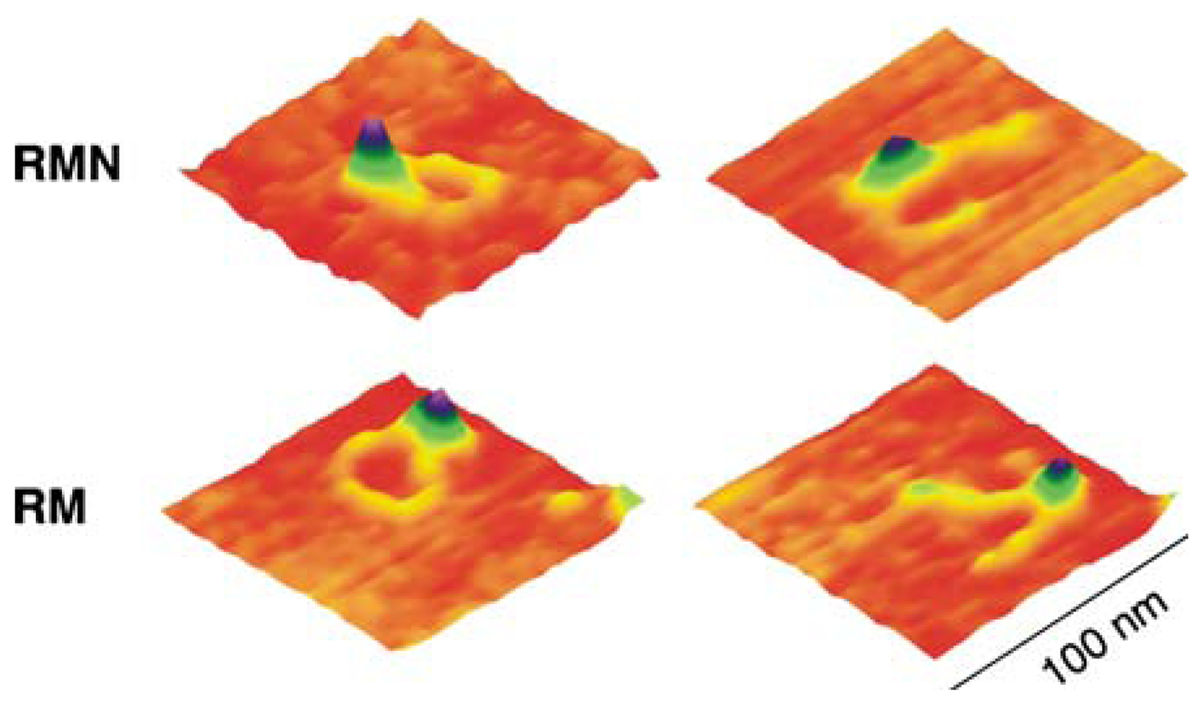
Scanning force microscopy images of purified human MR and MRN complexes. The large globular domain including the Rad50 ATPase heads, Mre11 and Nbs1, if present, is the high darker colored object from which the two 50 nm long coiled coils protrude. The top two images are MRN and the bottom two images are MR. The hook domains are not stable as the ‘closed’ (left images) and ‘open’ (right images) conformations are about equally prevalent. These approximately 100 nm×100 nm images are presented as tilted views to emphasize topography, color indicates height from 0 to 4 nm (red to purple)

Some speculation on the role of ATP in DNA binding and function can be considered on the basis of the similarities and differences in the molecular architecture of Rad50 complex, condensin, and cohesin (Fig. 2). Rad50 and the other SMC proteins have in common a very elongated overall structure due to an extended amino acid region encoding a coiled coil, up to 50 nm long. At one end of this elongated coiled coil, the juxtaposed N- and C-terminal domains form a functional ATPase. The other end of the coiled coil, the apex where the amino acid sequence turns back on itself, can be described in all of these proteins as an interaction domain (Fig. 2a). Rad50, condensin, and cohesin are all arranged in dimeric complexes of two elongated structures. The manner in which the elongated coiled-coil structures are connected and the resulting disposition of the ATPase domains differ between Rad50 and the SMCs cohesin and condensin (Fig. 2b). Cohesin and condensin dimerize by a stable interface at the globular ‘hinge’ domains located at the coiled-coil apex. This places the ATPase domains of the SMCs tethered at the ends of two long (and possibly flexible) coiled coils. In contrast, the smaller CxxC amino acid motif at the Rad50 coiled-coil apex is not a stable dimerization domain (Fig. 4). Two Rad50s are included in complexes by interaction with Mre11, which is a stable dimer alone and binds Rad50 along the coiled coils near the ATPase domain (Hopfner et al. 2001). Cohesin and condensin function together with partner proteins that interact at or near the ATPase domains. Inclusion of the SMC partner proteins in complexes modulates association of the ATPase domains and or ATPase activity, and can result in a ring-like structure in which both ends of the coiled coils are attached via protein–protein interactions (Haering et al. 2008). Rad50 functions together with Mre11, which defines the dimer interactions, and sometimes with Nbs1 in a yet undefined arrangement and stoichiometry.

Thus, all of these proteins have two long coiled coils that can be joined at either one end or both ends. The ATPase domains at one end of the coiled coils are expected to dimerize accompanied by ATP binding, as is characteristic of similar proteins in this family, the ABC transporters or RecA fold ATPases (Hopfner and Tainer 2003; Ye et al. 2004). This ATP binding-induced dimerization can involve either intra- or inter-monomer/complex interactions. Cohesin and condensin are stable dimers joined at their coiled-coil apexes. In the absence of other partner proteins, the relative orientation of their ATPase domains, at the ends of about 100 nm combined long coiled coils, is not necessarily defined. The two ATPase domains in one complex are held in high local concentration, whereby ATP binding is likely to involve their intra-complex dimerization. On the other hand, the Rad50 ATPase domains are held relatively closer together via the Mre11 dimer bound at an adjacent position on the coiled coils. This places the ATPase domains in close proximity but perhaps also in a constrained relative orientation. Depending on the arrangement of Rad50 and Mre11 at their interface, ATP-binding induced dimerization within the complex may be favored or even disfavored. The possibility that ATPase domain dimerization in Rad50 complexes involves inter-complex interactions is an intriguing possibility to be tested.

**Fig. 5:**
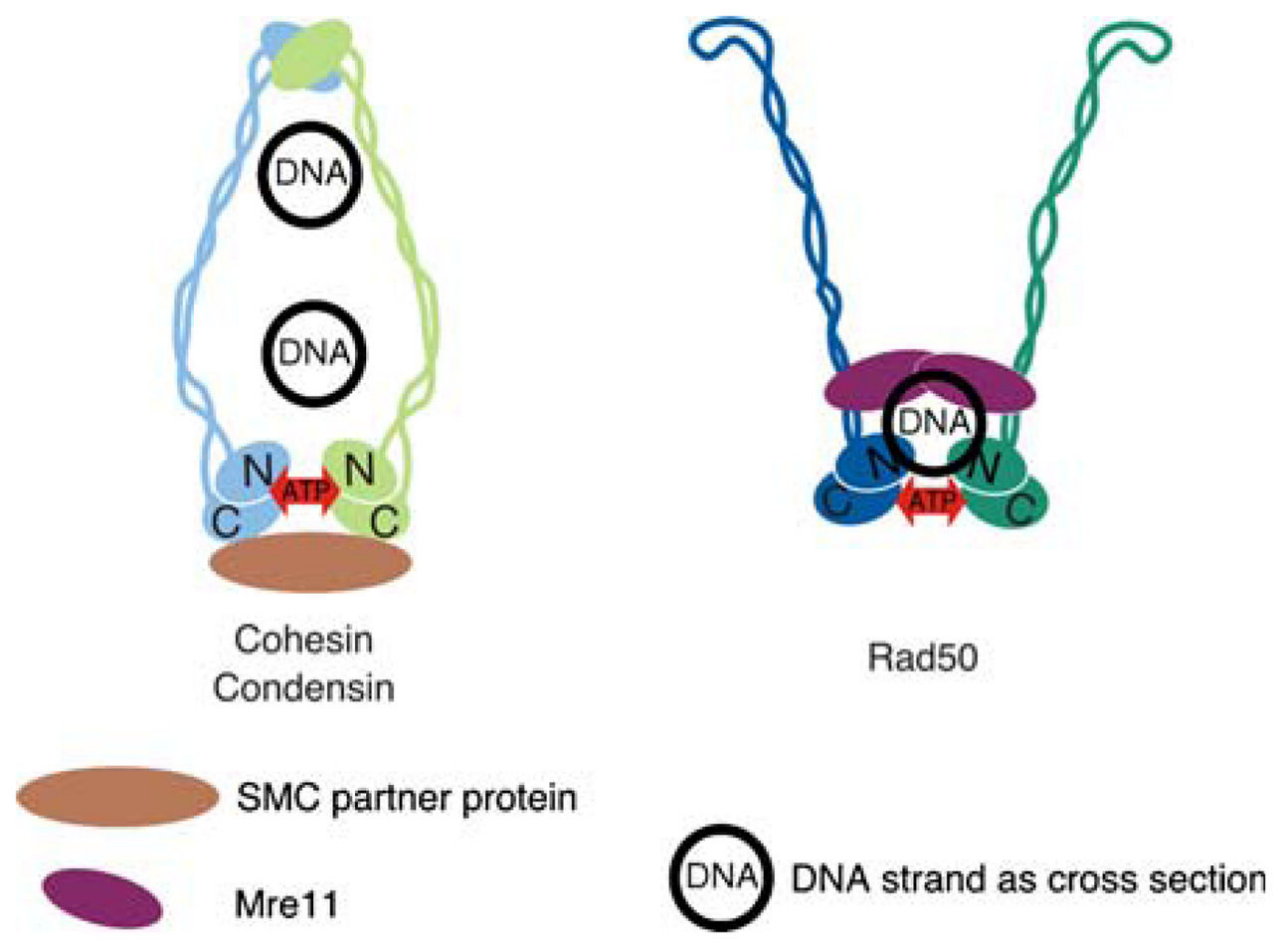
ATP-induced domain dimerization and DNA binding for SMC and Rad50 complexes. Cohesin and condensin organize DNA by trapping duplexes within a protein ring. Interaction with DNA can be controlled by closing and opening the protein ring. Both ATP binding-induced dimerization of ATPase domains and interactions with partner proteins are involved. In some SMC examples there are specific DNA binding sites, for others DNA is topologically linked to the protein complex. Rad50 complexes do not form large coiled-coil bound protein rings when bound to DNA. The conformation induced by DNA binding presumably inhibits intra-complex interaction of the coiled-coil apexes. DNA can bind to and be enzymatically processed by Mre11. If DNA binds on the surface of Mre11 in the orientation shown, then ATP binding-induced association of the head domains would be expected to modulate access to the Mre11 DNA-binding site or stability of the Mre11–DNA interaction. Currently the orientation of the Mre11 DNA-binding surfaces and the Rad50 globular domains is not known; one of the possible arrangements is shown. Here intra-complex ATPase site dimerization is shown. Association of ATPase sites between different complexes is also possible. This inter-complex dimerization may be favored between complexes bound near each other on DNA. The position of Nbs1 and its influence on complex architecture have not yet been determined. This simplified illustration is based on current knowledge. More complexity will surely be introduced when Nbs1 can be placed in the complex and possible DNA-binding sites on Rad50 are taken into account

The SMC cohesin and condensin complexes work in arranging DNA molecules by trapping one or more double helixes within a protein ring (Haering et al. 2002, 2008; Hirano 2005; Hirano and Hirano 2006) (Fig. 5). This arrangement does not require specific protein-DNA binding sites in order to work in organizing DNA molecules. Where specific binding sites have been identified or proposed they appear to be inside the potential protein ring. Thus, ring opening and closing via association of ATPase domains with each other or with partner proteins would control DNA access to these binding sites. By contrast, Rad50 complexes alone do not form stable rings via interactions at both ends of their coiled coils and are not expected to trap DNA within a large ring.

Conversely, DNA is an allosteric effector of MR, which changes the relative orientation of the coiled coils within a complex. Rad50 coiled coils are flexible, allowing their apexes to interact with each other (Fig. 4). Because this inter-complex binding between the hook domains is transiently observed in single complexes it appears to be relatively weak (Moreno-Herrero et al. 2005). Remarkably, once bound to DNA, the coiled coils become parallel to each other, an orientation that disfavors inter-complex interactions. In this way the hook domains of DNA bound MR(N) are poised to interact with those of other DNA bound complexes (Moreno-Herrero et al. 2005). Multiple MR(N) complexes bind to DNA, presenting a dense group of protruding hook domains. These provide multiple weak interacting partners to tether DNA bound by oligomers of MR (de Jager et al. 2001b; Hopfner et al. 2002). In contrast to other SMCs, inter-complex protein–protein interactions play an important role in this aspect of MR(N) function. Because it appears that individual hook–hook interactions are weak but that collectively many such interactions keep bound DNA molecules together, we describe MR as molecular Velcro for DNA.

The changes in orientation of Rad50 coiled coils upon DNA binding and DNA tethering by bound MR (N) oligomers are not influenced by ATP binding or hydrolysis (de Jager et al. 2001b, 2002; Moreno-Herrero et al. 2005). Thus ATP binding does not appear to affect interactions at the hook or hinge domain of MR(N). However ATP binding did affect the prevalence of large oligomeric MR complexes on DNAs with different end structures (de Jager et al. 2002). Because Mre11 has DNA end-specific activities, this could indicate ATP binding changes the orientation of the globular domains of the complex, specifically between Rad50 and Mre11. ATP binding-associated changes in the orientation of Rad50 ATPase domains could control access to DNA binding sites on Mre11. Depending on the exact architecture of the MR interactions, likely to be similar for human and yeast MR but so far best defined for pfRad50-Mre11 complex (Hopfner et al. 2001), intra-complex ATPase domain dimerization may trap DNA bound to Mre11 or prevent access to DNA binding sites on Mre11 (Fig. 5). Work with Rad50 alone suggests that a DNA-binding site is created by dimerization of ATP-binding domains. Whether two DNA-binding modes actually exist in intact complexes needs to be determined before their cooperation or competition for DNA can be addressed.

DNA binding is only the first step in the multiple activities of the Rad50 complexes that are essential for DNA break repair. The additional functionalities will inevitably involve changes in molecular architecture to promote new interactions. For instance, DNA tethering requires many protein complexes bound, perhaps cooperatively, to DNA. Thus factors that promote MR complex oligomerization and cooperative DNA binding are expected to be important regulators of this early step in DNA repair. Control of DNA end processing by MR(N) will likely involve modulating the access to Mre11 DNA-binding sites. In addition, ATM activation and cell-cycle signaling must require a specific molecular architecture of MRN complexes bound to DNA. We have focused here on details of the role of ATP in DNA binding by MR(N). For this first step there are still important unanswered questions. New information on the nature and control of inter- and intra-complex interactions and the dynamic arrangement of component proteins will provide valuable insight into how the relatively simple MR(N) molecular machine performs its many different jobs in DNA break repair.

## Abbreviations

ABC: ATP binding cassette
A-T: ataxia telangiectasia
ATLD: ataxia telangiectasia like disorder
ATM: the protein ataxia telangiectasia mutated
DSBs: DNA double-strand breaks
EMSA: electrophoretic mobility shift assay
MR: complex consisting of MRE11 and RAD50
MRN: complex consisting of MRE11, RAD50 and NBS1
MR(N): complex MR or MRN
MRX: complex consisting of the yeast proteins Mre11, Rad50 and Xrs2
NBS: Nijmegen breakage syndrome
NTP: nucleotide triphosphate
RPA: replication protein A
SFM: scanning force microscopy (also called AFM, atomic force microscopy)
SMC: structural maintenance of chromosomes
ssDNA: single-stranded DNA
